# The Role of Heart Rate, Body Temperature, and Respiratory Rate in Predicting Anastomotic Leakage following Surgery for Rectal Cancer

**DOI:** 10.1155/2021/8698923

**Published:** 2021-08-19

**Authors:** Jiajun Luo, Hongxue Wu, Yue Jiang, Yu Yang, Jingwen Yuan, Qiang Tong

**Affiliations:** Department of Gastrointestinal Surgery I Section, Renmin Hospital of Wuhan University, Wuhan 430060, China

## Abstract

**Objective:**

To explore the value of the heart rate, body temperature, and respiratory rate in the early prediction of anastomotic leakage after rectal cancer surgery.

**Methods:**

Clinical data from patients with rectal cancer who underwent anterior rectal resection in the Department of Gastroenterology, Renmin Hospital of Wuhan University, from January 2017 to December 2019 were collected and analyzed retrospectively. Based on the occurrence of anastomotic leakage after surgery, the patients were divided into two groups: those with and without anastomotic leakage. The quantitative values of the heart rate, body temperature, and respiration rate at day 7 postsurgery were compared between the two groups. The ROC curve was used to analyze their role in the early prediction of anastomotic leakage.

**Results:**

Among 441 patients with rectal cancer, 30 (6.81%) had clinical anastomotic leakage and were diagnosed at 7 ± 3 days postsurgery. Within 7 days postsurgery, the heart rate, body temperature, and respiratory rate in the anastomotic leakage group were higher than those in the nonanastomotic leakage group. The differences in heart rate (1-5 d), body temperature (2-7 d), and respiratory rate (1-7 d) were statistically significant (*P* < 0.05). The three ROC curves were drawn, respectively. The predictive value of the heart rate is greatest at days 2-3 postsurgery. The predictive value of the body temperature is greatest at days 4-6 postsurgery. The predictive value of the respiratory rate is best at days 1-4 postsurgery.

**Conclusion:**

The changes of vital signs (heart rate, body temperature, and respiratory rate) have a certain value in the early prediction of anastomotic leakage after rectal cancer surgery. Observation of postoperative vital signs at 7 days postsurgery is helpful for the early diagnosis of anastomotic leakage.

## 1. Introduction

Colorectal cancer is the third most common malignant tumor in the world and has the second highest mortality rate [1]. Surgery has long been considered the main treatment for colorectal cancer [2]. Anastomotic leakage is a major postoperative complication following colorectal surgery, and it is often associated with poor surgical outcomes [3]. Rectal cancer accounts for approximately 30% of colorectal cancer cases [4], and rectal surgery has a higher incidence of postoperative anastomotic leakage than colon surgery [5]. Anastomotic leakage after rectal surgery may adversely affect the morbidity, mortality, and prognosis of patients [6, 7]. Even with increased surgical proficiency, meticulous management of patients, and active attempts to avoid the occurrence of anastomotic leakage, the incidence of anastomotic leakage has not changed significantly [8]. Nowadays, due to the popularization of the enhanced recovery after surgery (ERAS) concept [9], the early diagnosis of anastomotic leakage is particularly important. For instance, early diagnosis can be used as a criterion for whether patients can be discharged early, which helps reduce the length of hospitalization and hospitalization costs for low-risk patients. This article is aimed at exploring the correlation of vital signs to the early prediction of anastomotic leakage following rectal cancer surgery by comparing the heart rate, body temperature, and respiratory rate within 7 days postsurgery.

## 2. Materials and Methods

This was a retrospective, single-center cohort study. We retrospectively analyzed the clinical data of patients undergoing anterior rectal resection for rectal cancer in the Department of Gastrointestinal Surgery of Renmin Hospital of Wuhan University from January 2017 to December 2019. This study was approved by the Ethics Committee of Renmin Hospital of Wuhan University.

### 2.1. Inclusion Criteria

All patients met the following requirements: (1) at least 18 years old; (2) the preoperative pathological diagnosis was confirmed to be rectal cancer, and the lower edge of the tumor was judged by colonoscopy or other examinations to be less than 15 cm from the anal margin; (3) the primary anastomosis is performed with anterior rectal resection; and (4) postoperative pathology confirms that the tumor was completely removed.

### 2.2. Exclusion Criteria

The patients who did not meet the inclusion criteria or meet the following characteristics were excluded: (1) palliative, Hartmann, or Miles surgery; (2) serious infection other than that caused by an anastomotic leak after the operation; (3) use of immunosuppressive drugs before surgery; (4) history of other malignancies; and (5) incomplete patient clinical data.

### 2.3. Diagnostic Criteria and Classification of Anastomotic Leakage

Diagnostic criteria are as follows: (1) the abdominal drainage tube drains out turbid pus-like or fecal-like matter, (2) the abdominal drainage tube has a sudden increase in drainage fluid or drainage of gas, (3) digital rectal examination reaches the stoma site defect, and (4) persistent fever, peritonitis, and reoperation found anastomotic defect.

The following are according to the 2010 International Study Group of Rectal Cancer (ISREC) definition and classification of anastomotic leakage after anterior rectal resection [10]: (1) grade A: subclinical anastomotic leakage, no clinical symptoms, and required no active therapeutic intervention; (2) grade B: manifested as abdominal pain; fever; purulent or scum-like drainage from the anus, drainage tube, or vagina; and white blood cells and C-reactive protein increased and required active therapeutic intervention but manageable without relaparotomy; and (3) grade C: manifested as peritonitis, sepsis, and other clinical manifestations of grade B anastomotic leakage and required relaparotomy.

### 2.4. Statistical Analysis

Statistical analyses were performed using SPSS Statistics 22.0 software and GraphPad Prism 8 v8.0.1. Data are presented as means ± standard error and were analyzed using Student's *t* test. Count data were expressed as percentages and were analyzed using *x*^2^ Fisher's test. The receiver operating curve (ROC) was used to evaluate the predictive effects of the postoperative heart rate, body temperature, and respiratory rate on anastomotic leakage after rectal cancer surgery.

## 3. Results

### 3.1. Population

A total of 441 patients with rectal cancer were included in the study according to the inclusion and exclusion criteria. All of them survived rectal surgery and were divided into two groups according to whether or not an anastomotic leakage occurred after the surgery.

Thirty-three patients (7.48%) had postoperative anastomotic leakage, which was diagnosed within 7 ± 3 days after the surgery. Among them, three cases (9.10%) were grade A leakage, fifteen cases were grade B leakage (45.45%), and fifteen cases (45.45%) were grade C leakage. The thirty patients with grade B and C leakage were regarded as the anastomotic leakage group. Characteristics of the patients are shown in Table 1. The postoperative hospital stay in the anastomotic leakage group was significantly longer than that in the nonanastomotic leakage group. Gender, diabetes mellitus, neoadjuvant CRT, albumin, and tumor distance from the anal margin were statistically significant in comparison between the two groups, while differences between the groups in the remaining assessed factors were not statistically significant.

### 3.2. Postoperative Trend of Heart Rate, Body Temperature, and Respiratory Rate

**Figure 1:**
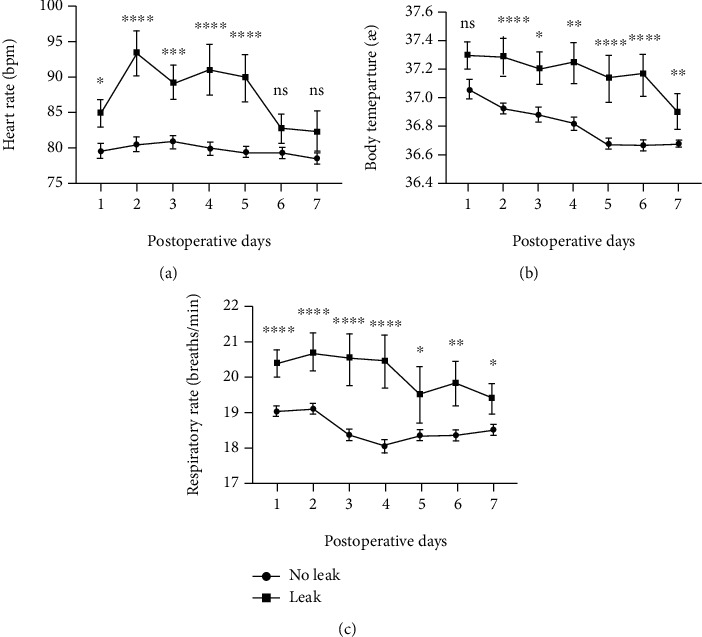
Mean levels of heart rate (a), body temperature (b), and respiratory rate (c) and relative error bars on days 1-7 after surgery.

The heart rate of the patients with anastomotic leakage was higher than those without leakage within 7 days postsurgery, and it was statistically significant within 1 to 5 days. From postsurgery day 2 onwards, the heart rate showed an overall downward trend, as shown in Figure 1(a).

The body temperature of patients with anastomotic leakage within 7 days after surgery was higher than those without anastomotic leakage, and it was statistically significant at days 2-7 postsurgery. The overall postoperative trend was a decrease in the body temperature, as shown in Figure 1(b).

The respiratory rate of patients with anastomotic leakage within 7 days after surgery was higher than those without anastomotic leakage, and it was statistically significant from 1-7 days postsurgery. The overall postoperative trend showed a downward trend in respiratory rate, as shown in Figure 1(c).

### 3.3. Predictive Effect of Postoperative Heart Rate, Body Temperature, and Respiratory Rate

**Figure 2:**
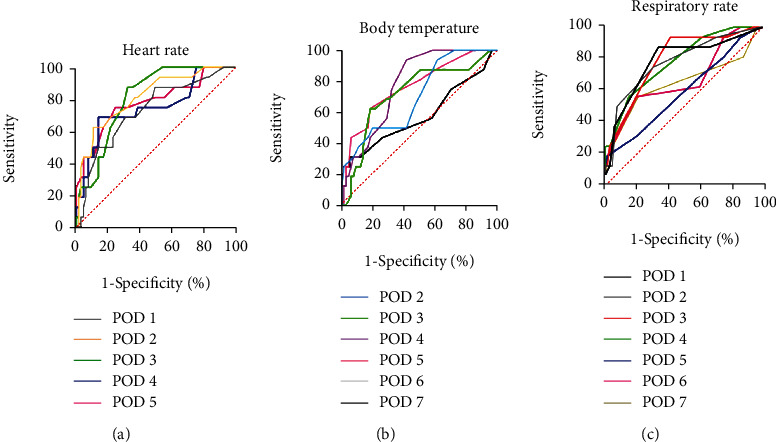
ROC curve analysis for heart rate (a), body temperature (b), and respiratory rate (c).

The postoperative heart rate, body temperature, and respiratory rate were drawn with ROC curves, as shown in Figure 2.

The area under the heart rate curve from 1 to 5 days after surgery was 0.73, 0.81, 0.81, 0.75, and 0.78, respectively, as shown in Figure 2(a). The prediction effect was best on days 2-3 postsurgery. The area under the curve was the largest on postsurgery day 2. When the heart rate was greater than 89 bpm, the sensitivity was 62.5% and the specificity was 89.2%.

The area under the body temperature curve from 2 to 7 days after surgery was 0.71, 0.72, 0.78, 0.77, 0.79, and 0.58, respectively, as shown in Figure 2(b). The prediction effect is best on days 4-6 postsurgery. The area under the curve was the largest on postsurgery day 6. When the body temperature is greater than 37°C, the sensitivity is 62.5% and the specificity is 85.3%.

The area under the respiratory rate curve from 1 to 7 days after surgery was 0.78, 0.78, 0.79, 0.78, 0.59, 0.67, and 0.64, respectively, as shown in Figure 2(c). The prediction effect is best on days 1-4 postsurgery. The area under the curve was the largest on postsurgery day 3. When the respiratory rate is greater than 20 breaths/min, the sensitivity is 62.5% and the specificity is 76.9%.

## 4. Discussion

Although the surgical techniques for colorectal cancer continue to advance, the incidence of postoperative anastomotic leakage has not been significantly reduced. According to reports in the literature, the incidence of anastomotic leakage after rectal surgery ranges from 3.1% to 13.7% [11]. In our study, the incidence of anastomotic leakage after radical resection of rectal cancer was 7.48%, which was consistent with reports in the literature. The average length of stay for patients with anastomotic leakage in this study was significantly higher than for those without anastomotic leakage. The occurrence of anastomotic leakage prolonged the patient's hospital stay and increased the economic burden on patients. The concept of ERAS has been gradually and steadily implemented in clinical practice, reducing hospitalization time, expenses, and psychological burden for most patients. However, there are also certain risks that may require patients with anastomotic leakage to undergo a second hospitalization or develop serious complications after discharge. Existing studies have reported many risk factors for anastomotic leakage, such as male gender, neoadjuvant CRT, anastomotic position, diabetes, and low albumin [12]. From the clinical data statistically compared in our study, male gender, diabetes, neoadjuvant therapy, low albumin, and distance from the anal margin ≤ 5 cm are all statistically significant risk factors for anastomotic leakage. In some studies, high BMI, low hemoglobin, large tumor diameter, and late pathological stage are also considered risk factors for anastomotic leakage, but in our study, these factors were not statistically significant. Numerous risk factors do not facilitate accurate prediction of the potential for anastomotic leakage after rectal surgery. Therefore, it is extremely urgent to develop diagnostic tools for early screening and the detection of anastomotic leakage in patients following rectal surgery. Diagnostic imaging and digital rectal examination are recognized procedures for the diagnosis of anastomotic leakage, but early diagnosis cannot be achieved. Many other laboratory tests such as C-reactive protein (CPR) [13], procalcitonin (PCT) [14], white blood cell (WBC) counts [15], and ascites cytokine [16] detection methods are currently under extensive research, but all still have their shortcomings. Other methods [17–19] that have been investigated have problems such as insufficient evidence or more complicated implementation. There is still no accepted method for the early diagnosis of anastomotic leakage in clinical practice.

Vital signs are the most accessible and noninvasive indicators, but their value in the early diagnosis of anastomotic leakage is still controversial in the current research [20]. Erb et al. [21] propose that abnormal vital signs such as fever and tachycardia are very common after rectal cancer surgery and are of no value for the early diagnosis of anastomotic leakage. In their study, abnormal vital signs were defined. They compared abnormal vital signs between patients with and without anastomotic leakage and concluded they showed no obvious predictive value. The study by Twohig et al. [22] found that 96.8% of patients had at least one abnormal vital sign after surgery, a common manifestation following surgery. A single vital sign alone cannot independently predict postoperative anastomotic leakage. However, our study found that not all patients exhibit abnormal vital signs following surgery. Patients with postoperative anastomotic leakage may have relatively slight changes in early vital signs compared with patients without anastomotic leakage. Stearns et al. [23] reported that changes in vital signs appeared early after surgery; more specifically, the heart rate, body temperature, and respiratory rate of patients with anastomotic leakage began to change 2 to 3 days after surgery. In our study, the heart rate and respiratory rate of patients with anastomotic leakage began to change from the first day after surgery, which may indicate the hemodynamic changes in the early stage of anastomotic leakage, and the body temperature began to change on day 2 postsurgery, which is consistent with other reports. Postoperative monitoring of vital signs is commonplace in clinical protocols. Compared with other laboratory and imaging tests, monitoring of vital signs has obvious advantages in the ease of acquisition but lacks diagnostic accuracy. For patients with abnormal vital signs, other relevant laboratory and imaging tests can be performed to confirm the diagnosis, so as to avoid the possibility of anastomotic leakage in patients who were not adequately monitored after early discharge and therefore susceptible to poor postoperative outcomes.

## 5. Conclusion

In conclusion, changes in postoperative vital signs have value for the early prediction of anastomotic leakage after rectal cancer surgery. Close observation of the patient's vital signs within 7 days after surgery is beneficial for the early diagnosis of anastomotic leakage.

## Figures and Tables

**Table 1:** Demographic and clinical data.

Parameter	Leak (*n* = 30)	No leak (*n* = 408)	*P* value
Gender (M (%))	23 (76.7)	222 (54.4)	0.018
Age (≥60 y, *n* (%))	24 (80.0)	273 (66.9)	0.139
Diabetes (*n* (%))	9 (30.0)	24 (5.88)	0.001
Cardiovascular disease (*n* (%))	8 (26.7)	84 (20.6)	0.430
BMI (≥25 kg/m^2^, *n* (%))	20 (66.6)	255 (62.5)	0.648
ASA score (3~4, *n* (%))	8 (26.7)	132 (32.4)	0.519
Hb (≤110 g/L, *n* (%))	5 (16.6)	60 (14.7)	0.770
ALB (≤35 g/L, *n* (%))	6 (20.0)	30 (7.35)	0.015
Surgical method (laparoscopy, *n* (%))	28 (93.3)	387 (94.9)	0.719
Prophylactic ileostomy (*n* (%))	9 (30.0)	118 (28.9)	0.900
Tumor distance from anal verge (≤5 cm, *n* (%))	13 (43.3)	96 (23.5)	0.015
Neoadjuvant CRT (*n* (%))	11 (36.6)	42 (10.3)	0.001
TNM stage (III~IV, *n* (%))	16 (53.3)	186 (45.9)	0.411
Tumor diameter (≥3 cm, *n* (%))	25 (83.3)	270 (66.2)	0.053
Postoperative hospital stay (days, *x* ± *s*)	24.5 ± 5.5	11.2 ± 4.1	0.001

Note: M: male; BMI: body mass index; ASA: American Society of Anesthesiologists; Hb: hemoglobin; ALB: albumin; CRT: chemoradiotherapy; TNM: tumor node metastasis.

## Data Availability

All data used and analyzed during the current study are available from the corresponding authors on reasonable request.
